# Six years beyond pediatric trauma: child and parental ratings of children’s health-related quality of life in relation to parental mental health

**DOI:** 10.1007/s11136-015-1002-y

**Published:** 2015-05-23

**Authors:** Kerstin Prignitz Sluys, Margaretha Lannge, Lennart Iselius, Lars E. Eriksson

**Affiliations:** Department of Molecular Medicine and Surgery, Karolinska University Hospital, L1:00, Solna, 171 76 Stockholm, Sweden; Department of Technology and Welfare, Red Cross University College, Stockholm, Sweden; Department of Pediatric Emergency Surgery, Astrid Lindgrens’ Children’s Hospital, Karolinska University Hospital, Stockholm, Sweden; Department of Molecular Medicine and Surgery, Karolinska Institutet, Stockholm, Sweden; Department of Neurobiology, Care Sciences and Society, Karolinska Institutet, Stockholm, Sweden; Department of Infectious Diseases Karolinska University Hospital, Huddinge, Sweden; School of Health Sciences, City University London, London, UK

**Keywords:** Injury, Trauma, Pediatric, Parents, PedsQL, Mental health, Depression, Health-related quality of life

## Abstract

**Purpose:**

To examine the relationship between child self-report and parent proxy report of health-related quality of life (HRQL) and how parents’ mental health status relates to the HRQL ratings 6 years after minor to severe injury of the child.

**Materials and methods:**

This cross-sectional cohort study was performed at a regional pediatric trauma center in Stockholm, Sweden. The PedsQL 4.0 versions for ages 5–7, 8–12, and 13–18 years were completed by 177 child–parent dyads 6 years after injury to the child. The parents also rated their own mental health through the mental health domain (MH) in the SF-36 Health Survey.

**Results:**

The children’s median age was 13 years (IQR 10–16 years), 54 % were males, and the median ISS was 5 (IQR 2–9). Most of the parents were female (77 %), born in Sweden (79 %), and half had university degrees. There was no statistically significant difference between child self-report and parent proxy report in any of the PedsQL 4.0 scales or summary scales. The levels of agreement between child self-report and parent proxy reports were excellent (ICC ≥ 0.80) for all scales with the exception of emotional functioning (ICC 0.53) which also was the scale with the lowest internal consistency in child self-report (*α* 0.60). Multiple regression analyses showed that worse parental mental health status correlated with worse child self-report and parent proxy report of children’s HRQL.

**Conclusions:**

Children and their parents’ reports on child’s HRQL were in agreement. Decreased mental health in parents was associated with lower scores on parent proxy reports and child self-reports of HRQL after injury. The current investigation highlights the possible relationship between parent’s mental health status and children’s HRQL long after an injury, which should be considered in future investigations and in clinical care.

## Introduction

Trauma is the most common cause of death and functional impairments among children and adolescents [1–3]. The currently held view is that traditional outcome measures, such as survival rates or presence of physical symptoms, are inadequate and do not capture the range of ways in which a patient may be affected by injury, treatments, and sequelae [4]. In the last decade, several authors have highlighted the importance of measuring health-related quality of life (HRQL) as an essential aspect of assessing outcome after injury [4–8].

HRQL instruments need to be multidimensional, consisting of physical, emotional, and social health dimensions based on the World Health Organizations (WHO) definition of each concept [4–9]. Because injury characteristics are heterogeneous, generic instruments are preferred and enable comparisons across multiple groups to facilitate understanding of how demographic variables or clinical groups differ in their reported HRQL scores [7, 9]. Disease-specific instruments can complement generic measures focusing on specific aspects of health with respect to particular disease or organ systems [7, 9].

Studies of children’s and adolescents’ HRQL face many challenges. One is that different researchers use different measuring instruments, which raises the question of whether the same health dimensions have been measured [9]. Another challenge arises from when and how the information was obtained. Most studies of HRQL of children after injury have been carried out within 2 years after injury and have relied on parents’ proxy reports. These studies have focused on different age ranges and injuries and have revealed rapid recovery during the first year after moderate to severe injuries, followed by a plateau phase during which any remaining disabilities remain more or less unchanged [10–17]. The few existing long-term follow-up studies have found that children continue to recover 5–10 years after moderate to severe injuries and a majority of them report HRQL scores similar to those of healthy peers [18–21]. Those studies have, however, either focused on specific injuries such as traumatic brain injuries [19, 21] and or had a specific focus on children with the most severe injuries [18, 20]. No long-term studies have been found representing the full spectra of injuries and injury severities found in a general pediatric population.

HRQL measures are by definition an individual’s perception of the effects of disease and treatment on their well-being [22]. The gold standard for measuring pediatric HRQL is self-report, as children have a unique awareness of their own health and earlier research has revealed that children as young as 5 years can self-report their HRQL [23, 24]. It is well documented in the literature that there are discrepancies between children’s self-report and parents’ proxy reports, where lower agreement have been found in subjective domains such as emotional and social functioning and higher agreement for objective domains such as physical functioning [25–27]. In studies where differences have been investigated it has been suggested that parents rate their children’s HRQL worse than the children themselves [27, 28]. There is also concern regarding the influence of parental distress and other related factors on parents’ perception of their child’s HRQL [14, 19, 28]. Most authors agree that it is important to include parents’ proxy report as a complement to child self-report as a secondary outcome measure. Moreover, there are situations where the child is unable to provide a self-report and parent proxy report is the only source of information [5–7, 27, 29]. A number of studies have examined children with traumatic brain injuries (TBI) and found that caring for children with TBI may have adverse effects on the home environment, potentially leading to parental mental health pathology, family dysfunction, and changes in the parent–child relationship [30–32]. There is, however, a knowledge gap regarding the situation in families after other types of injuries. Research is needed investigating the agreement between child self-report and parent proxy report of children’s HRQL and parental factors that may influence ratings of children’s HRQL.

In this study, we decided to use the Pediatric Quality of Life Inventory (PedsQL 4.0) since it assesses the domains outlined by the WHO, includes both child and proxy versions, has good psychometric properties, is widespread and easily interpretable, and recommended as a generic instrument for measuring children’s and adolescents’ HRQL after injury [4–8]. The purpose in the present study is to examine the relationship between child self-report and parent proxy report of children’s HRQL and how parents’ mental health status relates to ratings of child HRQL 6 years after the child had sustained a minor to severe injury.

## Methods and materials

### Participants

**Fig. 1:**
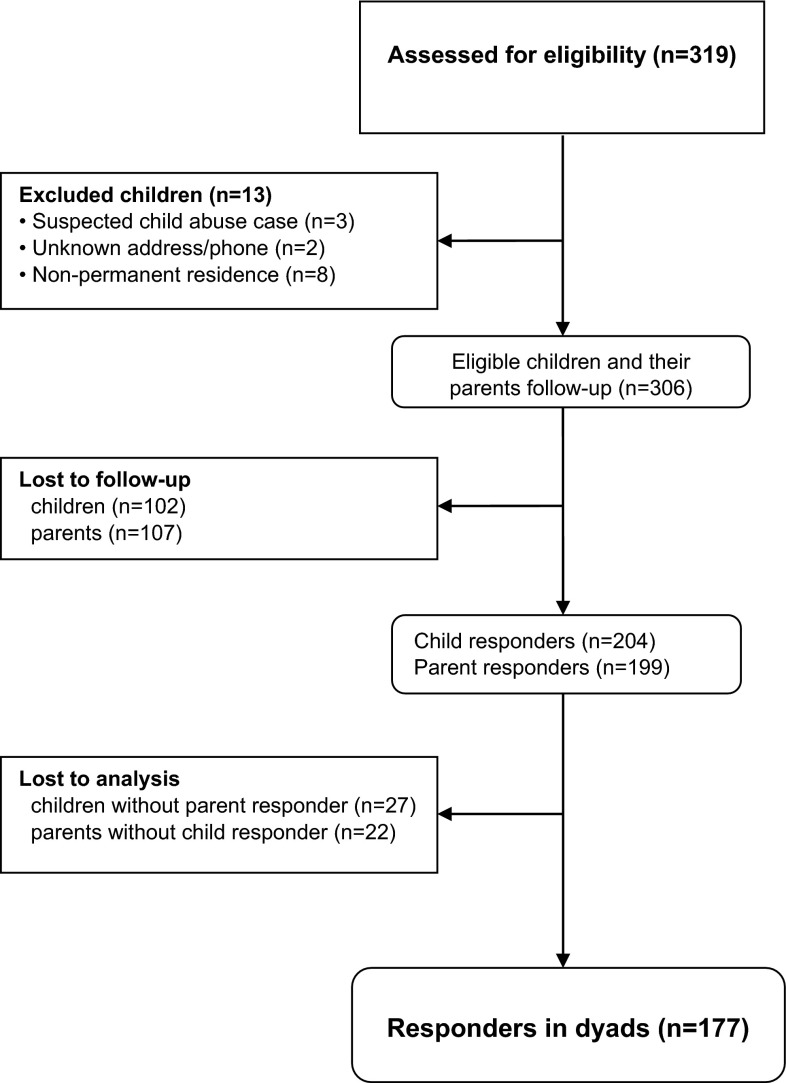
Flow diagram of the cohort

The data in this cross-sectional study derive from a series of studies on pediatric trauma outcome in the Stockholm region [33]. The current sub-study focuses on comparison of child self-report and parent proxy reports of HRQL 6 years after the injury event. Included in this current study are as follows: (1) children and adolescents 12 years or younger at the time of injury; with (2) minor to severe injuries (AIS ≥ 1); (3) who fulfilled the hospital’s trauma team activation criteria (see “Appendix”); and (4) were discharged alive after being admitted to the regional pediatric trauma center, Astrid Lindgren’s Children’s Hospital, Karolinska University Hospital (Stockholm, Sweden). Exclusion criteria were as follows: (1) suspicion of child abuse; (2) unknown address or phone number; (3) inability of child or parent to understand Swedish; and (4) non-permanent residence in Sweden. HRQL measurement instruments were administered to 306 children and their parents 6 years after injury. Two hundred and four children (reported elsewhere [33]) and 199 parents responded; of these, 177 were child–parent dyads (58 % of the original sample) and were included in this study. Figure 1 displays a flow chart of the cohort.

### Demographic and injury characteristics

Data were collected from the hospital trauma registry (Kvittra^®^, Combitech, Växsjö, Sweden) of Astrid Lindgren’s Children’s Hospital, Karolinska University Hospital. The registry holds information on demographics and injury characteristics such as age, sex, injury mechanisms, anatomical injury diagnoses, treatments, and patient outcomes. All children’s hospital medical records were re-reviewed for quality assurance [33]. The nature and severity of each injury were characterized according to the AIS-90 scale system [34]. The AIS classifies injuries by type and location and—with consideration of the child’s age—assigns severity in an ordinal scale from 1 (minor) to 6 (unsurvivable). To provide an overall severity score for children with multiple injuries, the Injury Severity Scale score (ISS) was computed. The ISS score is the sum of the squares of the three most severe AIS injuries sustained in three ISS body regions; scores range in an ordinal scale from 1 to 75, where 75 is unsurvivable [35].

### Questionnaire child self-report versus parent proxy report

We used the Swedish version of the PedsQL 4.0 generic core scales to measure the child’s HRQL [36]. The PedsQL encompasses 23 items that are divided into four domains: physical functioning (8 items), emotional functioning (5 items), social functioning (5 items), and school functioning (5 items). Child self-report includes versions for ages 5–7, 8–12, and 13–18 years where each version is essentially identical apart from some minor modifications in the wording based on the children’s ages. The parent proxy report version is constructed as a mirror of the child’s version and assesses the parent’s perceptions of their child’s HRQL [37].

In the present study, we used the child and proxy versions for ages 5–7 (young child), 8–12 (child), and 13–18 years (adolescent). The instructions ask how much of a problem each item has been for the child within the past month. The version for children’s self-report ages 5–7 years consists of a 3-point Likert scale with each response supported by a sad to a happy face scale, ranging from “not at all a problem” to “a big problem.” The versions for ages 8–12 and 13–18 consist of a 5-point Likert scale, ranging from “never a problem” to “almost always a problem.” Parent proxy report to each item is based on the 5-point Likert scale for all age groups. Raw score on each single item is transferred to a 0–100 scale (3-point Likert scales: 0 = 100; 2 = 50, 3 = 0 and 5-point Likert scales: 0 = 100; 1 = 75; 2 = 50; 3 = 25; 4 = 0), where higher scores reflect better perceived HRQL. Scale scores were calculated if there were responses to at least 50 % of the items in each respective scale, as recommended by the developer of the instrument [38]. The scales can also be combined into summary scales. The psychosocial health scores comprise the items included in the emotional, social, and school functioning scales (15 items) and the total health scores include the items of all four scales (23 items).

### Questionnaire for parents

The Swedish version of the SF-36 Health Survey was used as an outcome measure for parental mental health [36]. SF-36 is a generic short-form health survey consisting of 36 items divided into eight scales. The instrument has shown acceptable psychometric properties and is internationally widespread [39]. For the purpose of this study, we used the five-item mental health domain (MH) which is one of the eight scales of the SF-36.

The MH consists of the following questions: (1) Have you been a very nervous person? (2) Have you felt so down in the dumps that nothing could cheer you up? (3) Have you felt calm and peaceful? (4) Have you felt downhearted and blue? (5) Have you been a happy person? The response alternatives consist of 5-point Likert scale ranging from the “all the time” to “none of the time.” The ratings of the five items are transferred to a MH score with a possible range from 0 (worst) to 100 (best mental health). Additional questions were included in the questionnaire to gather information on parent demographic characteristics.

The MH scale score from this study was compared to an age-matched reference sample (*n* = 3429; ages 34–54 years) drawn from the Swedish SF-36 norm database (=8930) (Health Care Research Unit, Sahlgrenska University Hospital, Gothenburg, Sweden; *t* test for independent groups). The internal reliability coefficients for the MH scale used for comparison in this study had a mean Cronbach’s alpha coefficient of 0.86. [39].

### Procedures

Study procedures were reviewed and approved by the Regional Ethical Vetting Board (Stockholm). Six years after injury, children age 6–18 years at follow-up and their parents were contacted by mail with a cover letter, informed consent form, a questionnaire and a self-addressed stamped return envelop. Children 15 years of age or older were also contacted separately from their parents. Informed consent was obtained from all parents/guardians and children who were 15 years of age or older. Parents to children between the ages of 6 and 7 years were instructed to read the instructions and questions aloud to the child, while older children were instructed to answer the questions on their own. Parents were asked to complete the PedsQL 4.0 proxy version, the SF-36 questionnaire, and answer some additional questions.

### Statistical analysis

The software IBM SPSS Statistics 20.0 (IBM Corp., Armonk, NY, USA) was used for all the statistical analyses. Descriptive statistics were calculated for child and parent characteristics. Categorical variables are presented using frequencies and percentage, while continuous variables—if normally distributed—are presented as means and standard deviation (SD), or as median and interquartile range (IQR), if not normally distributed. Variables were considered significant at a *p* value of <0.05.

First, we determined the internal consistency for the PedsQL scales and the mental health scale in SF-36 by calculating Cronbach’s alpha coefficient of reliability. Second, related-samples Wilcoxon signed-rank tests were performed to test the differences in PedsQL scale and summary scores between child self-report and parent proxy report. Thirdly, t test for independent samples was computed between the age-matched mental health (MH) reference sample from the Swedish SF-36 norm database and the parents’ SF-36 MH scale scores. Fourthly, two-way mixed model intra-class correlations (ICC) with absolute agreement were computed between the children’s self-reported HRQL and the parent proxy reports to estimate levels of agreement. The strength of agreement was interpreted as <0.40 = Poor; 0.40–0.59 = Fair; 0.60–0.74 = Good; 0.75–1.00 = Excellent [40]. Lastly, step-wise multiple regression analysis was performed to find out how parents’ mental health status correlated with ratings of child HRQL in a model corrected for the variance of the child and parent background variables. The children’s current age, sex (1 = male, 2 = female), and injury severity score (ISS) were entered in the first step. The parent’s sex (1 = male, 2 = female), country of birth (1 = Sweden, 2 = any other country), and educational level (1 = lower than university, 2 = university degree) was entered in the second step. Finally, parent’s SF-36 mental health scores were entered in the third step. The effect size of the R^2^ changes in the third step was interpreted as small if 0.01, medium if 0.09 and large if 0.25 [41].

## Results

### Child demographic and injury characteristics

At follow-up 177 (58 %), child–parent dyads were obtained. The children’s median age at follow-up was 13 years (IQR 10–16 years), 96 (54 %) were males, and median ISS was 5 (IQR 2–9). Table 1 displays the children’s demographic and injury characteristics.

**Table 1:** Distribution of children’s demographic and injury characteristics

Characteristics	(*n* = 177)
Age at follow-up, [median (IQR)]	13 (10–16)
Male [*n* (%)]	96 (54.2)
ER only [*n* (%)]	69 (39.0)
PICU stay [*n* (%)]	43 (24.4)
Hospital length-of-stay [median (IQR)]	2 (1–3)
Blunt trauma	171 (96.6)
Mechanism of injury	
Traffic-related events [*n* (%)]	68 (38.4)
Fall [*n* (%)]	71 (40.1)
Other [*n* (%)]	38 (21.5)
Location of injury	
Head (cranium and brain) [*n* (%)]	81 (45.8)
Moderate (AIS 2) [*n* (%)]	61 (34.5)
Severe (AIS 3) [*n* (%)]	13 (7.3)
Serious (AIS 4–5) [*n* (%)]	7 (4.0)
Extremities (AIS ≥ 2) [*n* (%)]	41 (20.1)
ISS score [n (%)]	
≤8	116 (65.5)
9–15	46 (26.0)
≥16	15 (8.5)

IQR, interquartile range; ER, Emergency Department discharged within 24 h; PICU, pediatric intensive care unit; AIS, Abbreviated Injury Scale scores (1–6); extremities, upper and lower extremities; ISS, Injury Severity Scale scores (1–75)

#### Parent demographic characteristics

Of the responding parents, 137 (77 %) were females, 139 (79 %) were born in Sweden, and 89 (50 %) had university degrees.

### Agreement between parent proxy and child self-report HRQL

Internal consistency of PedsQL 4.0 for parent proxy and child self-report exceeded the minimum reliability standard of *α* 0.70 required for group comparisons [42, 43]. The only scale that did not reach the recommended level was emotional functioning (*α* 0.60) in the child self-report. There was no statistically significant difference between child self-report and parent proxy report in any of the PedsQL 4.0 scales or summary scales (Table 2). The ICC estimates of agreement between the children’s self-reported HRQL and the parent proxy reports were excellent (≥0.80) with the exception of the scale emotional functioning were the level was fair (0.53). (Table 2).

**Table 2:** Children’s PedsQL scores reported by child and parent

PedsQL	Child’s report (*n* = 177)		Parent’s proxy report (*n* = 177)		p**	ICC
	Median (IQR)	α*	Median (IQR)	α*		
Total scale score	91.3 (84.7–95.6)	0.88	90.2 (82.6–95.6)	0.90	0.239	0.83
Psychosocial health	90.0 (80.0–96.6)	0.84	90.0 (80.0–95.8)	0.89	0.269	0.80
Physical health	93.7 (87.5–100)	0.76	96.8 (87.5–100)	0.79	0.712	0.83
Emotional functioning	90.0 (75.0–100)	0.60	85.0 (70.0–100)	0.83	0.081	0.53
Social functioning	100 (90.0–100)	0.82	100 (90.0–100)	0.85	0.761	0.86
School functioning	90.0 (70.0–100)	0.77	90.0 (65.0–100)	0.86	0.185	0.82

PedsQL, Pediatric Quality of Life Inventory; ICC, intra-class correlation coefficient

* Cronbach’s *α*

** Child’s report versus parent’s proxy report. Wilcoxon signed-rank test

#### Hierarchical multiple regression

When comparing the parents’ SF-36 MH scale scores [mean 79.1 (SD 20.3)] with the MH age-matched reference group [mean 80.7 (SD 19.2)], there were no significant difference (*p* = 0.146). Two sets of hierarchical multiple regression analyses were performed to investigate whether the parent’s MH correlated with child and parent ratings of children’s HRQL. The two sets of models contained seven predictors and were entered in the three steps presented in Table 3. Adding parental mental health (MH) in the third and final step caused a statistically significant *R*^2^-change for all PedsQL scales and summary scales with the exception of the child self-reported scale emotional functioning. The statistically significant *R*^2^-changes of the third step were of medium effect size in all models except for proxy ratings of school functioning where it was of small effect size. This means that parental MH was positively correlated with the children’s self-rated and parents’ proxy rated HRQL scores when the variance of the child’s and parent’s background variables already had been taken into account.

**Table 3:** Hierarchical multiple linear regression of the relationship of parents’ SF-36 mental health scores on child self-report and parent proxy report of PedsQL 4.0

Predictors	Total scale score		Psychosocial health		Physical health		Emotional functioning		Social functioning		School functioning	
	Child	Proxy	Child	Proxy	Child	Proxy	Child	Proxy	Child	Proxy	Child	Proxy
*Independent variable/model summary*												
Step 1												
Child age, *β*	0.144	−0.053	0.205**	−0.043	−0.021	−0.062	0.259**	−0.127	0.303***	0.101	−0.042	−0.028
Child ISS, *β*	0.074	−0.016	0.066	−0.018	0.064	−0.004	−0.059	0.051	0.139	0.009	0.054	0.001
Child sex, *β*	−0.029	0.077	0.006	0.098	−0.086	0.018	−0.030	0.119	−0.019	0.062	0.069	0.044
*R* ^2^	0.027	0.009	0.045	0.012	0.013	0.004	0.073**	0.033	0.108***	0.014	0.009	0.003
*F*	1.527	0.582	2.616	0.764	0.708	0.264	4.374	2.175	6.726	0.882	0.516	0.167
*df* (regression; residual)	3;166	3;189	3;166	3;189	3;166	3;189	3;166	3;189	3;166	3;189	3;164	3;182
Step 2												
Child age, *β*	0.134	−0.073	0.187*	−0.059	−0.016	−0.087	0.246**	−0.137	0.276***	0.071	−0.048	−0.036
Child ISS, *β*	0.075	−0.016	0.069	−0.013	0.061	−0.015	−0.057	−0.056	0.124	0.004	0.073	0.021
Child sex, *β*	0.007	0.119	0.040	0.146*	−0.092	0.033	−0.012	0.130	0.000	0.114	0.111	0.096
Parent sex, *β*	0.088	0.079	0.100	0.064	0.031	0.092	0.040	0.060	0.217**	0.141*	−0.024	−0.019
Parent born, *β*	0.069	−0.207**	−0.141	−0.170*	0.073	−0.233**	−0.120	−0.076	−0.218**	−0.276***	−0.037	−0.093
Parent education, *β*	0.137	0.165	0.192*	0.203**	0.000	0.037	0.089	0.050	0.155*	0.222**	0.203*	0.218**
*R* ^2^-change	0.031	0.069**	0.063*	0.067**	0.006	0.061**	0.021	0.011	0.115***	0.132***	0.038	0.050*
*F*-change	1.793	4.618	3.812	4.481	0.350	4.015	1.288	0.717	8.052	9.592	2.155	3.182
*df* (regression; residual)	3;163	3;186	3;163	3;186	3;163	3;186	3;163	3;186	3;163	1;185	3;161	3;178
Step 3												
Child age, *β*	0.09	−0.194	0.082	−0.173*	−0.139	−0.193**	0.208**	−0.227**	0.180*	−0.049	−0.160*	−0.107
Child ISS, *β*	0.113	0.017	0.101	0.018	0.099	0.013	−0.046	−0.031	0.153*	0.037	0.105	0.040
Child sex, *β*	0.003	0.124	0.049	0.151*	−0.081	0.037	−0.008	0.133	0.008	0.120	0.116	0.105
Parent sex, *β*	0.120	0.122	0.126	0.104	0.062	0.130*	0.049	0.093	0.241***	0.184**	0.002	0.005
Parent born, *β*	0.53	−0.072	−0.038	−0.043	0.193*	−0.116	−0.083	0.024	−0.125	−0.143*	0.070	−0.019
Parent education, *β*	0.24	0.028	0.097	0.074	−0.111	−0.083	0.055	−0.052	0.069	0.086	0.108	0.144
Parent MH, *β*	0.433***	0.470***	0.363***	0.442***	0.427***	0.411***	0.132	0.352***	0.330***	0.467***	0.374***	0.260**
*R* ^2^-change	0.140***	0.166***	0.099***	0.147***	0.137***	0.126***	0.013	0.093***	0.081***	0.163***	0.104***	0.050**
*F*-change	28.300	40.552	20.124	34.977	26.185	28.896	2.357	19.911	18.921	43.737	19.555	9.919
*df* (regression; residual)	1;162	1;185	1;162	1;185	1;162	1;185	1;162	1;185	1;162	1;185	1;160	1;178

^β^Standardized beta coefficient

PedsQL, Pediatric Quality of Life Inventory; SF-36, short-form health survey; Child ISS (1–75); Child sex, 1 = male, 2 = female; Parent sex, 1 = male, 2 = female; Parent born, 1 = Sweden, 2 = any other country; Parent education, 1 = educational level lower than university, 2 = educational level university degree; Parent MH, Parent SF-36 Mental Health score

* *p* < 0.05; ** *p* < 0.01; *** *p* < 0.001

##### Child’s characteristics as predictors in addition to parental MH in the final models (step 3)

Higher current age of the child predicted higher self-reported scores in emotional functioning (*p* < 0.01) and social functioning (*p* < 0.05), whereas higher age predicted lower self-reported scores in school functioning (*p* < 0.05). Conversely, higher current age of the child predicted lower proxy scores in emotional functioning (*p* < 0.01), physical health (*p* < 0.01) and psychosocial health (*p* < 0.05). Higher injury severity scores (ISS) predicted higher self-reported scores in social functioning (*p* < 0.05), and female sex of the child predicted higher proxy scores in psychosocial health (*p* < 0.05).

##### Parent characteristics as predictors in addition to parental MH in the final models (step 3)

Female sex of the parent predicted higher scores in both child self-reports and proxy reports of social functioning (child report *p* < 0.001; parent report *p* < 0.01) and proxy reports of physical health (*p* < 0.05). Parents born in another country predicted lower proxy scores in social functioning (*p* < 0.05) and higher self-report scores in physical health (*p* < 0.05). Parent’s educational level did not predict child HRQL.

## Discussion

In this study, we used the PedsQL 4.0 instrument to determine the relationship between child and parent proxy ratings of children’s and adolescents’ HRQL as assessed by 177 child-parent 6 years after an injury to the child. We also used the SF-36 health survey instrument to explore the parents’ mental health status. Hierarchical multiple regression analyses were used to investigate the correlation of the parent’s mental health status to both the child’s and the parent’s rating of the child’s HRQL. To our knowledge, this is the first study that has investigated the relation of parent’s mental health status on child and parent ratings of children’s HRQL after injury.

The main finding of the present study is that a low score for parent’s mental health status was the strongest predictor of poorer children’s HRQL in all domains in parent proxy reports. It was also the strongest predictor of poorer HRQL as reported by children themselves. However, the relationship may be either way or bidirectional; parental mental health may influence children’s HRQL as well as children’s HRQL influencing parental mental health. Two earlier studies have explored parental mental health and the relation between child and parent ratings of children’s HRQL. Panepinto et al. [44] in a study using PedsQL 4.0 to determine the HRQL of children with sickle cell disease found that parents with lower mental health status proxy rated HRQL scores that were significantly lower than their children’s self-reported HRQL. The authors suggested that the children may have adjusted to their level of functioning and therefore reported better HRQL compared to their parents ratings. In contrast, Vance et al. [45] in a study of children with acute lymphoblastic leukemia found that parents who were more depressed had children that self-reported poorer HRQL, the parent’s depression was not related to the proxy report of the child’s HRQL. In the same study, illness stressors and perceived vulnerability were correlated with significantly poorer parents’ proxy ratings. [45] Vance and colleagues suggested that parents are better able to hide stress, but unable to hide more pervasive feelings of depression. The differences between our results and the results of these two studies may be related to time of follow-up, method of data collection, different diagnosis, and cultural differences. Our results could also reflect parents’ knowledge concerning their child’s experiences, health and well-being. This has been showed by Varni et al. [46] in a study where there were higher agreements in domains that were of clinical importance to the child’s health problem. The possible bidirectional relation in our result could also be related to well-known research findings that parents are affected by children’s exposure to traumatic events and that their responses impact children’s reaction to trauma. [47] Traumatic events such as a high-speed vehicle crash can cause instability in a child’s life, which has been found to be associated with a range of outcomes impacting development and affecting cognitive abilities, school achievements, social skills, and behavior. [47] Earlier follow-up studies of injured children have demonstrated that caregiver distress, socioeconomic difficulties, and family burdens are associated with lower parent proxy report scores of the child’s HRQL after injury. [14, 19, 28, 48–50] Wade and colleagues [48] found in a study of pediatric trauma that social relationships are important for parents’ psychological adjustment regardless of injury. A study by McCarthy et al. [14] found that unhealthy family functioning prior to the child’s injury, single-parent household, and not being covered by insurance were associated with worse parent proxy reports of children’s HRQL. In addition, Aitkin et al. [49] found that burden in families after pediatric trauma was greater when health care need was unmet. This has also been found in a study by Limond et al. [50] on children with spinal cord injury (SCI) where 45 % of the parents perceived that they did not receive enough support after discharge from acute care hospital after their child’s injury. The parents in Limond and colleagues [50] study reported significantly lower scores on their child’s HRQL. These findings suggest that children’s HRQL may be better in families that have better economic and psychosocial conditions and that such conditions facilitate adjustment after pediatric injuries. Further research is necessary to reveal causal relationship between parents’ mental health and child and parents ratings of children’s quality of life.

We found no discrepancies between the parents’ proxy report and the children’s self-report of the child’s HRQL. The only PedsQL scale that showed a tendency to significant difference in ratings was emotional functioning, where parents tended to rate their children’s function worse than the children themselves. The level of agreement between child and parent proxy ratings of children’s HRQL report was strong in all scales with the exception of emotional functioning which was also the scale with the lowest internal consistency. In a study by Glaser et al. [51], the authors claimed a higher level of agreement (however, not statistically different) in child and parent ratings of children’s HRQL when the questionnaire was mailed and completed at home compared to completion at a clinical setting. The authors state that this might be explainable by collusion between children and their parents, but another factor that they also mention is that the completion of a questionnaire at home in familiar surroundings may provide a more accurate reflection of the child’s HRQL. The authors also found higher agreement between child and parent proxy ratings compared to child and physician proxy ratings and child and physiotherapist proxy ratings, suggesting that proxies who have the greatest contact with the child respond more comparably with the child [51]. In a review study by Upton et al. [52], the authors found no differences in parent proxy reports and children’s self-report agreement depending on method and place of data collection. In the present study, the questionnaires were sent to the children’s and parents’ home addresses with instructions to avoid collusion and so enhance agreement, but there was no control over how the questionnaires were filled out. Therefore, we cannot rule out that the relationship between child and parent reports could have been affected by the method of data collection, both positively and negatively. Further research is needed to identify factors that may influence levels of agreement in child and proxy ratings of children’s HRQL.

In our multiple regression models, older children were found to report higher HRQL in emotional and social functioning. Conversely, parents of older children reported lower scores in emotional functioning, psychosocial health and physical health. These findings are somewhat in line with several previous investigations of child and parent reports on HRQL. For example, Achenbach et al. [25] found that parents are more adept at assessing a child’s externalizing problems (e.g., aggression and conduct) compared to internalizing problems (e.g., anxiety and depression). Eiser and Morse [27] have suggested that this could be applied to parents being more prone to rate the child’s HRQL on the basis of visible domains such as physical functioning than on less visible domains such as emotional or social functioning. We also found that parents of female children reported higher scores in psychosocial health.

Children with more severe injuries reported better social functioning in their HRQL. To our knowledge, this finding has not been described earlier. In a Swedish qualitative study of adolescents with spinal cord injury (SCI), Augutis et al. [53] parents and peers were found to have formed an important support network around the injured child. Parents acted as advocates and containers for sadness, frustration and anger, and friends acted as promoters of activities and identity development. It was perceived that healthcare providers did not make sufficient use of this network [53]. It is possible that children with more serious injuries receive better support from their social network. Further studies are needed in this area to investigate the impact of social support from family, friends and others regarding help to cope and adjust after different injuries.

Mothers as proxy reporters dominate most studies. In a study by Waters et al. [54] of healthy school children, the mother’s self-reported HRQL significantly influenced the proxy report on their children’s HRQL. The author did not find this association with fathers [54]. In the study by Vance et al. [45] of children with cancer, it was found that children who self-reported poorer HRQL had mothers who were more depressed. In the present study, 77 % of the parent responders were females, and if the proxy reporter was female, this predicted an increase in both child and parent reports of social functioning and in parent reports of physical health, but the strongest predictor of parents’ ratings of their children’s HRQL was the parents’ mental health status.

### Strengths and limitations

One strength of the present study is the long-term follow-up. Earlier studies have shown that children’s recovery trajectory continues 5–10 years after injury, indicating that follow-up investigations should go beyond 5 years [18–21]. Another strength is that the population derives from a complete cohort from a well-defined population and geographical area (Stockholm region).

Some limitations of this study should be noted. First, the cross-sectional design does not allow controlling for pre-injury HRQL and other confounding variables such as recurrent injuries or other health problems. Furthermore, we did not have access to data to control for personality characteristics, family dynamics, and resources. Additional exploration of these issues is clearly merited. Secondly, 42 % of the cohort was lost to follow-up causing selection bias which potentially limits the generalizability of the findings. We recommend the readers to interpret the results with caution. Responders and non-responders were comparable with regard to demographic characteristics, but non-responders had less severe injuries and were more often discharged home from the emergency department than the responders. These factors may have influenced the recall of the injury event and the interest in participating, as reported elsewhere (in manuscript). A reminder to non-responders would probably have helped achieve a higher response rate, but such procedures were not permitted by the ethical review board.

## Conclusions

Children and their parents reported concurrent PedsQL 4.0 scores. Results indicate that poor parental mental health has a possible relationship on both the child’s and the parent’s ratings of children’s HRQL. The present findings can in several ways contribute to future research and clinical management. First, subsequent investigations may consider taking the measurement of parents’ mental health status into account in future research of children’s HRQL since it appears to be a significant factor in interpreting the results. Longitudinal studies investigating parents’ mental health and children’s HRQL in parallel are also essential to further reveal the causal relationship between parents’ mental health and children’s quality of life. Finally, we suggest developing well-validated risk assessment tools that can be feasibly implemented in clinical practice for diverse injury events that will help identify the high-risk youth and families who are in need of clinical services. Early detection of children with poor HRQL and parents who suffer from poor mental health seems to be important not only for the long-term health and recovery of the injured child but also for the parents’ and families’ well-being.

## Appendix

Trauma team activation criteria at Astrid Lindgren Children’s Hospital, Karolinska University Hospital, Stockholm, Sweden.

*Physiological criteria*

Respiratory impairment

Hypotension

Altered consciousness or neurological impairment

*and/or*

*Anatomical criteria*

Penetrating injuries to head, neck, torso, and extremities proximal to elbow and knee

Two or more long bone fractures

Pelvic fractures

Paralysis after trauma mechanism

Amputation proximal to wrist and ankle

Burn injuries or hypothermia combined with other trauma mechanism

Near drowning combined with other trauma mechanism

Flail chest

*and/or*

*Mechanism of injury*

High-speed crash

≥70 km/h with restraint use or air bag

≥50 km/h without restraint use or air bag

Vehicle entrapment, rollover

Ejection from vehicle, death in same vehicle

Pedestrian/bicyclist struck by vehicle

Fall of > 3 meter

Crush injuries torso
